# Natural phenolics as multitarget antimicrobials for food preservation: mechanisms of action

**DOI:** 10.1016/j.fochx.2025.103056

**Published:** 2025-09-20

**Authors:** Lei Zhao, Ya Zhou, Weiguo Yue, Qingshan Shen, Jingxuan Ke, Yanli Ma, Lifang Zhang, Hua Bian

**Affiliations:** a Henan Key Laboratory of Zhang Zhongjing Formulae and Herbs for Immunoregulation, Zhang Zhongjing College of Chinese Medicine, Nanyang Institute of Technology, Changjiang Road 80, Nanyang 473004, Henan, China; b Nanyang City Hospital of Traditional Chinese Medicine, Nanyang, Henan 473000, China

**Keywords:** Natural phenolics, Antibacterial mechanisms, Reactive oxygen species, Membrane disruption, DNA binding

## Abstract

Natural phenolics are emerging as promising clean-label antimicrobials, yet evidence for their multitarget mechanisms remains scattered. This review synthesizes 158 studies (2013–2025) on *Escherichia coli*, *Staphylococcus aureus*, and related pathogens. Three converging antibacterial targets are identified: ROS generation (72 % of phenolics), membrane disruption (58 %), and DNA interaction (41 %). Compounds such as bisdemethoxycurcumin, gallic acid, thymol, and Epigallocatechin gallate (EGCG) act across all targets, reducing bacterial counts by up to 4 log CFU/mL at ≤2 × MIC. A cascade mechanism is proposed: ROS triggers lipid peroxidation, weakening membranes, enhancing phenolic uptake, and accelerating DNA damage. Food matrix factors (pH, fat, water activity, microbiota) can suppress efficacy by up to 90 %. Emerging delivery strategies—nanoemulsions, biopolymer capsules, and active films—partially restore function. This review integrates molecular insights with food system data, offering a practical framework for designing robust phenolic-based antimicrobials.

## Highlights

• Natural phenolics disrupt bacteria via ROS, membrane damage, and DNA binding.
• A cascade model explains how multitarget synergy enhances antimicrobial efficacy.
• Experimental data verify BDMC's DNA interaction and oxidative membrane stress.
• Food matrix interference and delivery strategies are critically reviewed.
• Insights guide the clean-label design of phenolic-based food preservatives.

## Graphical abstract

**Figure:**
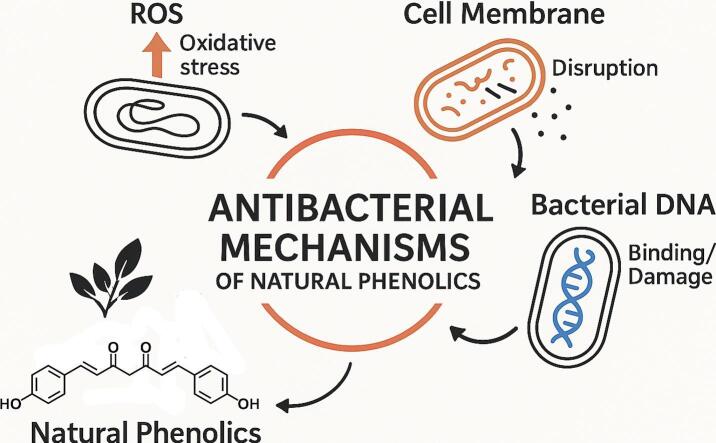

## 1 Introduction

Foodborne spoilage and pathogen control continue to drive demand for clean-label strategies in the food industry (Tian et al., 2024). Natural phenolics are widely investigated because they act on multiple antibacterial targets and are compatible with diverse food matrices (Yin et al., 2025). Mechanistic work to date has centred on reactive oxygen species (ROS) generation, membrane disruption, and DNA interaction, with representative compounds—including bisdemethoxycurcumin (BDMC) and epigallocatechin gallate (EGCG)—tested against major foodborne pathogens such as *Escherichia coli* and *Staphylococcus aureus* (Zhao et al., 2025). Recent studies further highlight the need to integrate multitarget mechanisms into food preservation strategies.

From 2023–2025, several studies have clarified how these targets interact rather than act in isolation. Phenolics that modestly elevate ROS can precipitate proton-motive force (PMF) collapse and permeability increases, thereby amplifying downstream damage to nucleic acids and metabolic networks; delivery formats (e.g., nanoemulsions, biopolymer microcapsules) further bias target priority by modulating compound localisation and release kinetics (Zhai et al., 2025). In parallel, comparative omics and spectroscopic readouts link DNA-level engagement with transcriptional changes in stress-response and virulence pathways, reinforcing a multitarget, cascade-like view of phenolic action (Ciamponi et al., 2022).

However, most existing reviews still emphasise individual mechanisms—such as oxidative stress (De Rossi et al., 2025), membrane permeability changes (Zhong et al., 2023), or DNA damage (Rispo et al., 2024). This single-target lens neither reflects the complexity of bacterial physiology nor accounts for the interconnections between cellular responses. Notably, synergistic or sequential effects—for example, ROS-induced membrane destabilization or enhanced DNA accessibility following permeability disruption—are rarely addressed within a unified framework (Gray et al., 2024).

**Fig. 1:**
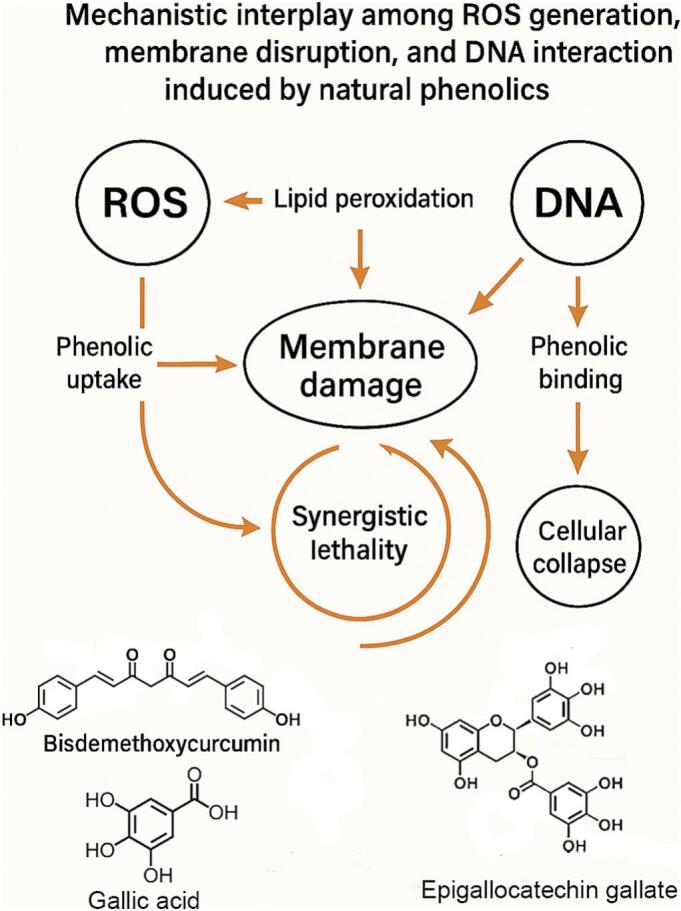
Mechanistic interplay among reactive‑oxygen-species (ROS) generation, membrane disruption and DNA interaction induced by natural phenolics. ROS produced by redox-active phenolics initiate lipid peroxidation, weakening the cytoplasmic membrane and increasing compound uptake. The resulting membrane damage accelerates intracellular ROS accumulation and facilitates phenolic access to genomic DNA. Direct groove binding or ROS-driven oxidative lesions destabilize DNA, activate SOS responses and exhaust antioxidant reserves, closing a positive-feedback loop that amplifies cellular collapse. Representative multitarget phenolics—BDMC, gallic acid and EGCG—are shown to illustrate structure–function links.

**Fig. 2:**
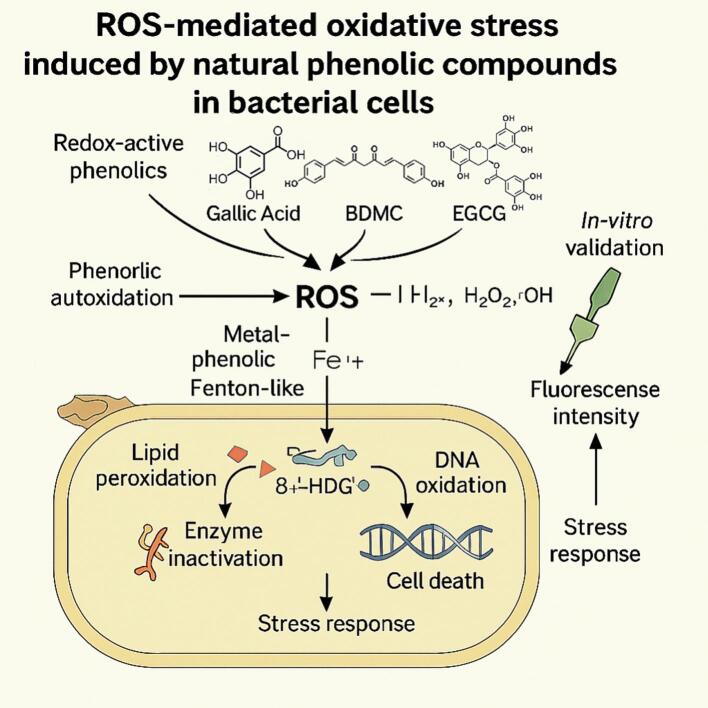
Reactive‑oxygen-species (ROS) pathway triggered by natural phenolics in bacterial cells. Redox-active phenolics—illustrated here with gallic acid, BDMC and EGCG—undergo autoxidation or metal-phenolic (Fenton-like) reactions to generate O₂^−^, H₂O₂ and •OH. The accumulated ROS initiate lipid-peroxidation cascades that weaken the membrane, oxidise key enzymes, and produce DNA lesions (e.g., 8-OHdG), collectively driving stress responses and cell death. DCFH-DA fluorescence is shown as the standard in vitro validation of ROS elevation.

To address this gap, this review integrates mechanistic evidence across ROS, membrane, and DNA targets under food-relevant conditions, outlines emerging mechanisms (protein thiol oxidation, quorum-sensing interference, and energy disruption via PMF/ATP), and discusses how delivery strategies can shift target priority. Representative compounds—BDMC, gallic acid, and EGCG—are used to illustrate structure–function relationships relevant to food safety applications. A schematic overview of the multitarget pathways is presented in Fig. 1 to guide the reader through the subsequent sections. (See Fig. 2.)

## 2 Literature search strategy

To ensure a comprehensive and methodologically sound synthesis of the antibacterial mechanisms of natural phenolic compounds, a structured literature review was conducted. Given the complexity and overlap among potential mechanisms—including ROS induction, membrane destabilization, and nucleic acid interference—a targeted search strategy was essential for delineating and evaluating evidence across these domains. This section outlines the rationale, scope, and technical implementation of the literature search, which formed the basis for the tripartite classification and mechanistic analysis presented in 3, 4.

### 2.1 Review scope and strategy

This review integrates mechanistic evidence on the multitarget antibacterial mechanisms of natural phenolic compounds, focusing on agents that induce ROS, disrupt membranes, and interact with DNA. We conducted a target-oriented literature search (Web of Science, Scopus, and PubMed; 2013–2025; English-language; peer-reviewed primary studies and reviews), prioritizing food-relevant endpoints. Particular emphasis was placed on major foodborne pathogens, including *Escherichia coli* and *Staphylococcus aureus*, and on representative compounds such as BDMC with documented multitarget effects. Foundational pre-2013 works were cited only when essential for mechanistic context. Search strategy: Web of Science, Scopus and PubMed (2013–2025; English; peer-reviewed). We included plant-derived phenolics with mechanistic readouts against food-relevant bacteria and excluded non-phenolics, non-bacterial or purely clinical contexts.

### 2.2 Databases and search terms

Three multidisciplinary databases—Web of Science Core Collection, Scopus, and PubMed—were consulted to ensure comprehensive coverage of the relevant literature. Searches were restricted to English-language, peer-reviewed articles published between January 2013 and March 2025; the final update was performed on 30 June 2025. Combined Boolean queries were applied as follows:

(“polyphenol” OR “phenolic compound”)

AND (“antibacterial” OR “antimicrobial”).

AND (“reactive oxygen species” OR ROS OR “oxidative stress”).

AND (“membrane damage” OR “membrane integrity” OR “permeability”).

AND (“DNA binding” OR “nucleic acid interaction” OR “genotoxic”).

Supplementary searches incorporating terms such as “BDMC,” “curcuminoid,” “lipid peroxidation,” and “fluorescence displacement” were executed to capture compound-specific and method-oriented studies.

### 2.3 Inclusion and exclusion criteria

**Inclusion criteria**

(1) Investigation of antibacterial effects exerted by plant-derived phenolic compounds.

(2) Presentation of mechanistic evidence involving at least one of the following targets: reactive oxygen species, cell membrane integrity, or bacterial DNA.

(3) Employment of experimental methods appropriate for mechanism verification (e.g., DCFH-DA assay, propidium iodide staining, DNA-binding spectroscopy).

(4) Use of bacterial strains relevant to food safety, such as *Escherichia coli*, *Staphylococcus aureus*, *Listeria monocytogenes*, or other common foodborne pathogens.

**Exclusion criteria**

(1) Studies centred on non-phenolic natural products (e.g., alkaloids, peptides).

(2) Articles addressing solely antioxidant or anti-inflammatory activity without antibacterial evaluation.

(3) Reviews, editorials, conference abstracts, patents, or papers lacking mechanistic data.

### 2.4 Data screening and selection flow

Initial searches retrieved 1230 records (Scopus 604; Web of Science 393; PubMed 233). Automated duplicate removal eliminated 200 entries, leaving 1030 unique records for title and abstract screening. Of these, 800 records were excluded for irrelevance to the inclusion criteria. The remaining 230 articles underwent full-text assessment, during which 88 were excluded owing to insufficient mechanistic evidence or non-foodborne bacterial models. Consequently, 142 publications were included in the qualitative synthesis.

A PRISMA-style flow diagram summarising this process is provided in Supplementary Fig. S1. The curated corpus forms the basis for the mechanistic classification (Section 3), target-specific discussion (4.1, 4.2, 4.3), and evaluation of functional performance in food matrices (Section 5).

## 3 Classification of multitarget antibacterial mechanisms

While the antibacterial effects of natural phenolics have been extensively reported, the mechanistic landscape remains fragmented due to the diversity of experimental models and endpoints employed. Many studies focus narrowly on a single mode of action, such as ROS generation or membrane disruption, without evaluating potential synergistic or sequential interactions across targets. To address this gap and provide a cohesive analytical structure, this section introduces a classification system that groups antibacterial mechanisms by their primary cellular targets. This approach enables clearer comparison across studies and highlights the multitarget potential of specific phenolic compounds, laying the groundwork for detailed mechanistic analysis in Section 4.

### 3.1 Review rationale

Natural phenolic compounds rarely confine their antibacterial action to a single cellular target. Instead, they trigger a constellation of disruptive events that collectively overwhelm bacterial defence systems (Gao et al., 2025). To compare and synthesise this diverse literature, a target-oriented framework is required. The present review therefore adopts a tripartite classification that groups mechanistic evidence under the three most consistently demonstrated targets—ROS, the cytoplasmic membrane, and bacterial DNA.

### 3.2 Definitions of the three Core targets

The antibacterial activity of natural phenolic compounds is generally mediated through three interconnected cellular targets: ROS, the cytoplasmic membrane, and bacterial DNA.

First, ROS-mediated oxidative stress arises from intracellular accumulation of superoxide anions (O₂^−^), hydrogen peroxide (H₂O₂), or hydroxyl radicals (•OH), often initiated by phenolic redox cycling or metal chelation (Liu et al., 2023). These reactive species cause non-specific oxidation of lipids, proteins, and nucleic acids, leading to broad cellular dysfunction (Juan et al., 2021). *Operationally, ROS generation is best evidenced by convergent assays:* generic dyes (e.g., DCFH-DA) should be complemented by EPR spin-trapping (e.g., DMPO) and genetically encoded redox sensors (roGFP2/HyPer) to resolve oxidant identity and redox state under food-relevant conditions (Akter et al., 2021; Fuloria et al., 2021). Inclusion of scavengers/enzymes (thiourea, catalase, superoxide dismutase) and oxygen-limited controls helps attribute growth inhibition to ROS rather than unrelated stressors (Ye et al., 2021).

Second, membrane disruption involves either direct insertion of phenolic compounds into the phospholipid bilayer or secondary damage via lipid peroxidation (Hossain et al., 2022). These processes compromise membrane integrity, alter permeability, dissipate membrane potential, and trigger leakage of cytoplasmic contents (Ganapathy et al., 2021). *Quantitatively, loss of membrane function is defined as* Δψ depolarisation and permeability increase: DiSC3(5)/DiOC2(3) report depolarisation (Buttress et al., 2022); NPN uptake (outer membrane, Gram-negatives) and PI/SYTOX Green influx (cytoplasmic membrane) assess permeability (Tajuelo et al., 2023). High-resolution imaging—confocal/TIRF time-lapse, AFM (roughness/stiffness), and cryo-SEM/TEM—provides orthogonal confirmation, while Laurdan generalized polarisation reports changes in lipid order/packing (Parthasarathi et al., 2025).

Third, DNA interaction and inhibition occur through minor-groove binding, electrostatic attraction, or ROS-induced oxidative lesions (Sanchez-Martin et al., 2022). These interactions interfere with bacterial DNA replication and transcription, resulting in impaired genomic stability and cell death (Lima et al., 2023). *Direct engagement is supported by spectroscopic and biophysical readouts:* UV–Vis/CD signatures (e.g., hyperchromicity at ≈260 nm; characteristic CD ellipticity changes) (Tang, 2025), thermal melting (ΔT_m_) and fluorescence intercalator-displacement with EB (intercalator) and DAPI/Hoechst (minor-groove) (Gupta et al., 2023). Binding thermodynamics/kinetics can be resolved by ITC (ΔH, ΔS, K_d_) and SPR (k_on_/k_off_) (Gupta et al., 2023) while EMSA and transcriptional readouts of SOS-response genes (e.g., *recA*, *lexA*) verify functional consequences (He et al., 2025).

These three targets are not independent; rather, they are mechanistically interrelated. For example, ROS generation can initiate lipid peroxidation, which weakens the cell membrane and facilitates phenolic uptake (Wang, Wang, et al., 2023). Enhanced intracellular concentration further amplifies oxidative stress and increases access to the nucleoid, where phenolics may induce DNA damage (Tatipamula & Kukavica, 2021). This interdependence forms a positive-feedback loop contributing to bacterial inactivation and is schematically illustrated in Fig. 1. To minimise dye/spectral artefacts from coloured phenolics, measurements should include dye-only/compound-only controls, inner-filter/turbidity corrections (e.g., front-face geometry or dilution), and parallel viability readouts; results are best interpreted from convergent assays rather than a single probe (Ceresa et al., 2021).

### 3.3 Representative phenolics and supporting evidence

Four well-characterised phenolic compounds—BDMC, gallic acid, thymol and EGCG—illustrate how distinct chemical scaffolds converge on the three core antibacterial targets described above. Key findings are summarised in Table 1 and briefly discussed here to provide mechanistic context for 4.1, 4.2, 4.3.

**Table 1:** Representative natural phenolic compounds with multitarget antibacterial activity: associated mechanisms, experimental methods, and principal findings.

Compound	Dominant Target(s)	Key Methods Employed	Principal Findings	Refs.*
BDMC	ROS, Membrane, DNA	DCFH-DA assay; protein leakage; EB/DAPI displacement	ROS ↑ >150 % (vs. control); 52 % protein loss at 2× MIC; groove- and structure-modifying DNA binding	(Alhasawi et al., 2022)
Gallic acid	ROS-driven	EPR; TBARS assay; bacterial killing assays	Light-exposed GA generates hydroxyl radicals (•OH) via ROS, damaging bacterial membranes & lipids, leading to cell death; TBARS confirms lipid peroxidation in *E. coli*	Hadidi et al. (2024); Nakamura et al. (2012)
Thymol	Membrane-centred	PI and DiBAC₄(3) staining; membrane potential microscopy	Rapid membrane depolarisation in *S. aureus*; ATP leakage; ROS-independent action	Li et al. (2022)
EGCG	DNA & ROS	CD; SPR; DCFH-DA assay	EGCG exhibits DNA minor-groove binding with K_D ∼1–5 μM (CD and SPR assays); concurrently induces measurable ROS increase in bacterial cells	Tang, 2025

BDMC. Curcuminoid derivatives such as BDMC couple a highly conjugated β-diketone chromophore with moderate hydrophobicity, enabling simultaneous oxidative and physical damage. In a standardized *E. coli* model, BDMC raised intracellular ROS by >150 % (DCFH-DA fluorescence), reduced soluble cytoplasmic protein by 52 % at 2 × MIC—consistent with membrane leakage—and interacted with genomic DNA through minor-groove binding, as indicated by ethidium-bromide and DAPI displacement assays (Dai et al., 2022). These data confirm that BDMC engages all three cellular targets in a single treatment regime.

Gallic acid. The trihydroxybenzoic acid nucleus of gallic acid confers strong redox activity. Electron-paramagnetic-resonance spectra show rapid generation of hydroxyl radicals on exposure to physiological oxygen, while TBARS measurements reveal extensive lipid peroxidation in *E. coli*, linking ROS formation to membrane injury and bactericidal action (Hadidi et al., 2024; Nakamura et al., 2012). Because gallic acid is highly polar, membrane binding is secondary; oxidative stress remains its dominant mode of action.

Thymol. As a monoterpenoid phenol, thymol partitions into the phospholipid bilayer within minutes. DiBAC₄(3) flow-cytometry analysis demonstrated marked membrane-potential collapse (*p* < 0.005) in *Staphylococcus aureus* after six hours at 500 μg mL^−1^, and propidium-iodide staining confirmed loss of membrane integrity. No significant ROS surge was detected, indicating a mainly ROS-independent membrane-disruption pathway (Li et al., 2022).

EGCG. The galloylated catechin EGCG targets nucleic acids while exerting moderate redox pressure. UV–Vis titration and circular-dichroism spectroscopy showed selective binding to G-quadruplex DNA with low-micromolar affinity, generating hyperchromicity and minor blue shifts characteristic of groove or π–π stacking interactions (Ali et al., 2023; Chen et al., 2024; Tang, 2025). Although not a potent pro-oxidant, several investigations report modest ROS elevation accompanying EGCG treatment (De Rossi et al., 2025; Zhong et al., 2023), suggesting that oxidative stress may reinforce its DNA-centred mechanism.

Together these examples underscore the structural diversity of natural phenolics and illustrate how individual compounds may favour one cellular target while still influencing the others—a theme explored in detail in the following sections.

### 3.4 Mechanistic crosstalk and synergy

Experimental evidence indicates a positive feedback loop among the three targets. ROS initiates lipid peroxidation, compromising the bilayer and accelerating phenolic influx. Increased intracellular concentration magnifies ROS formation and facilitates DNA access. Conversely, DNA oxidation triggers the bacterial SOS response, depleting antioxidative reserves and exacerbating oxidative stress. These interactions explain why multitarget phenolics often exhibit greater potency than the sum of their individual effects (Kamat & Badrinarayanan, 2023). Beyond the three core targets, additional mechanisms may operate in parallel or upstream (Section 3.5), further broadening the antibacterial landscape of phenolics under food-relevant conditions.

### 3.5 Emerging targets and research gaps

*Protein thiol oxidation.* Beyond ROS-, membrane-, and DNA-directed actions, catechol- and enol-bearing phenolics can undergo auto-/enzyme-catalysed oxidation to o-quinones that covalently trap protein cysteine thiols, forming Cys–quinone adducts and perturbing redox homeostasis and proteostasis in bacteria. Recent food/biomacromolecule studies demonstrate rapid thiol capture by oxidised polyphenols and map these adducts at cysteine sites, providing a biochemical basis for thiol-centric toxicity (Zhang et al., 2024). In microbes, thiol-redox vulnerability (e.g., OxyR/Trx/Prx systems) modulates sensitivity to electrophilic phenolics, suggesting that quinone-driven cysteine modification may synergise with oxidative stress to impair redox enzymes and membrane proteins (Ouyang et al., 2023). To quantify target engagement under food-relevant conditions, we recommend thiol-reactive chemoproteomic workflows (iodoTMT/OxICAT) combined with redox-sensors (roGFP2/HyPer) and in situ labelling (maleimide probes) to rank site-specific cysteine oxidation in key bacterial proteins (Li et al., 2023).

*Quorum-sensing (QS) interference.* Multiple phenolics attenuate virulence by disrupting AI-1/AI-2 signalling: flavonoids such as quercetin competitively bind LasR/RhlR or inhibit LasI/RhlI, down-regulating QS regulons and biofilm formation in *Pseudomonas aeruginosa* (Ren et al., 2024); green-tea EGCG lowers autoinducer output and QS-controlled genes in *Salmonella enterica*, diminishing colonization in vivo (Cheng et al., 2024). Standardized QS assays, including bioluminescent *Vibrio harveyi* AI-2 reporter strains (e.g., BB170) and AHL biosensor systems such as *Chromobacterium violaceum* CV026 (Jiang et al., 2022), together with quantitative PCR of QS regulatory genes (Jiang et al., 2022), are broadly recommended to accompany antimicrobial assays. For instance, Santos et al. (2021) demonstrated the use of phenolic compounds to inhibit QS in foodborne bacteria, distinguishing QS effects from antimicrobial activity via violacein-based biosensors. Separately, Belanger and Hancock (2021) outlined methods to assess antimicrobial activity under simulated physiological environments. However, to date, no study has yet combined both approaches to quantify the minimal QS-inhibitory concentration under realistic food matrix conditions such as pH, water activity, or fat content.

*Energy disruption* via *PMF collapse/ATP synthase inhibition.* Phenolic monoterpenes such as thymol and carvacrol act as proton exchangers that dissipate ΔpH/Δψ, collapsing the proton-motive force (PMF) and depleting ATP pools in foodborne bacteria (Mihaylova et al., 2025); delivery systems that raise local membrane concentrations (e.g., nanoencapsulation) can bias target priority toward energy failure (Hajibonabi et al., 2023). In parallel, stilbenoids such as resveratrol inhibit the bacterial F₁F₀-ATP synthase—sensitizing *Staphylococcus aureus* to aminoglycosides by up to 16-fold—and thereby define a complementary “energy-checkpoint” vulnerable to phenolic interference (Mackieh et al., 2023). We recommend a common panel—membrane-potential dyes (DiOC2(3)/DiSC3(5)), intracellular ATP assays (luciferase), and PMF reporters—run alongside viability/biofilm endpoints to quantify the contribution of energy uncoupling within the multitarget cascade.

*Practical gap.* Delivery-system effects (nanoemulsions, biopolymer microcapsules) should be explicitly linked to target bias using co-localization imaging and release-kinetics modeling under real food storage scenarios (see Section 2), enabling quantitative ranking of emerging targets relative to ROS, membrane, and DNA pathways.

## 4 Overview of core antibacterial mechanisms

Building on the tripartite classification outlined in Section 3, this section analyses three principal antibacterial targets of natural phenolics—ROS, cell membranes, and bacterial DNA—highlighting their individual roles and potential synergies. Each mechanism is explored in terms of its biological relevance, experimental validation, and compound-specific contributions. Special attention is given to compounds such as BDMC, which display multitarget properties supported by in vitro and spectroscopic evidence. The mechanisms are discussed in separate subsections (4.1–4.3) to highlight both their independent functions and interactive synergies.

### 4.1 ROS-mediated oxidative stress

ROS are widely recognized as key contributors to the antimicrobial mechanisms of natural phenolic compounds. These highly reactive molecules—including O₂^−^, H₂O₂, and •OH—can cause oxidative damage to bacterial membranes, proteins, and nucleic acids, ultimately leading to cell death (Ali et al., 2023).

Phenolic compounds such as bisdemethoxycurcumin (BDMC) have been reported to induce intracellular accumulation of reactive oxygen species (ROS) in bacterial cells, either via redox cycling or by impairing endogenous antioxidant defenses such as catalase and superoxide dismutase (Chen et al., 2024; Laha et al., 2022; Rizvi et al., 2023). Under standardized conditions (2023–2024), representative data from our laboratory (raw data available upon request) showed that BDMC elicited a dose-dependent rise in intracellular ROS in *Escherichia coli*, with DCFH-DA fluorescence increasing by >150 % at 40 mg mL^−1^ versus the untreated control (*p* < 0.001). These observations, together with published reports, support oxidative-stress induction as a key component of BDMC's antibacterial mechanism. The trend of ROS elevation was closely aligned with the minimum inhibitory concentration (MIC) of BDMC (10 mg mL^−1^). At concentrations ≥10 mg mL^−1^, ROS levels rose sharply, suggesting a causal relationship between oxidative stress and bacterial inactivation. This trend is consistent with previous reports on polyphenols where ROS induction preceded membrane disruption (Laha et al., 2022).

Mechanistically, ROS-induced damage may compromise bacterial cell membranes via lipid peroxidation, enhance permeability, and disrupt redox balance. These changes can trigger leakage of cytoplasmic contents and interfere with essential metabolic processes (Zhang et al., 2023). In our analysis, the increase in ROS was accompanied by membrane damage indicators, which are discussed in Section 4.2.

Although DCFH-DA remains a commonly employed probe for ROS detection, it lacks species specificity and is prone to interference from extracellular artefacts. Furthermore, the intracellular localization of BDMC and its capacity to initiate ROS generation at specific subcellular sites have yet to be clearly elucidated (Ruijter et al., 2024). Future studies employing ROS-specific probes, live-cell imaging, or electron paramagnetic resonance (EPR) techniques may offer improved mechanistic resolution (Menezes et al., 2024).

In summary, ROS-mediated oxidative stress represents a core mechanism by which phenolic antimicrobials such as BDMC exert their bactericidal activity. Understanding this oxidative pathway provides mechanistic insight and may guide the development of synergistic antibacterial systems targeting redox vulnerability in foodborne pathogens.

### 4.2 Cell membrane disruption

Disruption of bacterial cell membrane integrity is a well-established antibacterial mechanism, particularly among natural phenolic compounds. The cytoplasmic membrane is essential for maintaining ionic gradients, metabolic fluxes, and intracellular homeostasis; its destabilization leads to leakage of vital components and irreversible cell damage (Nourbakhsh et al., 2022).

**Fig. 3:**
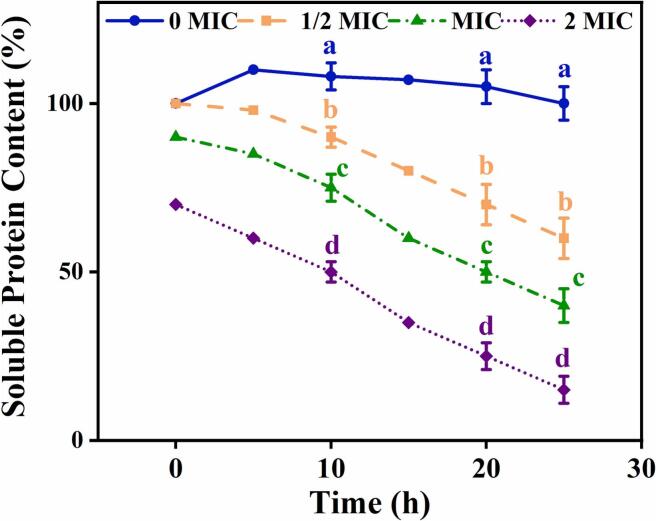
Dose- and time-dependent decrease in soluble protein content in *Escherichia coli* after exposure to bisdemethoxycurcumin (BDMC). Treatments were 0, ½, 1, and 2 × MIC BDMC with sampling up to 25 h. Values are mean ± SD (*n* = 3). Different lowercase letters (a–d) within each time point denote significant differences among treatments (one-way ANOVA with Tukey's HSD, *p* < 0.05). MIC, minimum inhibitory concentration.

Phenolic compounds such as BDMC exert membrane-disruptive effects via multiple converging pathways, including direct interaction with phospholipid bilayers, lipid peroxidation triggered by ROS, and alterations in membrane potential or permeability. Our recent experimental observations revealed that BDMC treatment resulted in substantial protein leakage in *E. coli*, with a reduction of over 50 % at 2 × MIC within 12 h (*p* < 0.001), consistent with pronounced membrane compromise (see Fig. 3).

Membrane disruption. Representative data from our laboratory (raw data available upon request) demonstrate rapid membrane depolarization and permeabilization in BDMC-treated *Escherichia coli*, evidenced by DiSC3(5) quenching within minutes, propidium iodide (PI) uptake, and leakage of K^+^ and A260-absorbing intracellular material. These biophysical signatures are characteristic of phenolic-induced envelope damage and have been reported for structurally related phenolics/essential-oil components in foodborne bacteria (Mráz et al., 2025; Noui Mehidi et al., 2024). Together with the ROS data above, these findings support a model in which BDMC compromises envelope integrity and collapses the proton-motive force, thereby contributing centrally to its antibacterial activity.

**Fig. 4:**
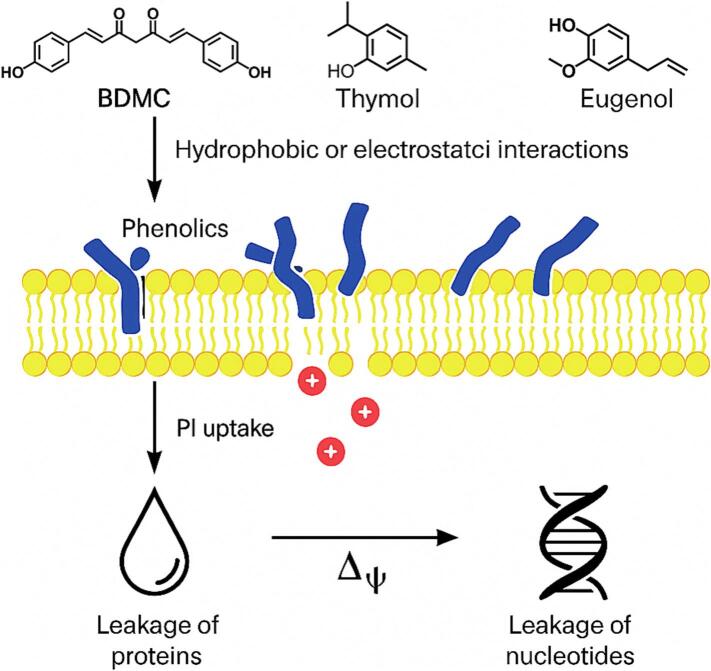
Schematic illustration of bacterial membrane disruption by natural phenolic compounds. Natural phenolics such as BDMC, thymol, and eugenol integrate into the bacterial phospholipid bilayer via hydrophobic or electrostatic interactions. These interactions lead to structural destabilization and transient pore formation, resulting in elevated membrane permeability and dissipation of membrane potential (Δψ). Consequent leakage of intracellular components, including proteins and nucleotides, is commonly verified by propidium iodide (PI) staining and protein leakage assays. This membrane-targeted mechanism significantly contributes to the antibacterial activity of multitarget phenolic compounds.

To contextualize these findings, Fig. 4 illustrates the proposed mechanism by which BDMC and structurally similar phenolics (e.g., thymol, eugenol) compromise membrane integrity. Their hydrophobic or electrostatic interactions with lipid bilayers may promote PI uptake, trigger loss of membrane potential (Δψ), and result in leakage of proteins and nucleotides. This model aligns with previous studies demonstrating that polyphenols destabilize bacterial membranes via combined physical insertion and oxidative perturbation (Czerkas et al., 2024; De Rossi et al., 2025).

Although soluble protein leakage provides indirect evidence of membrane disruption, it may also reflect secondary effects such as cell lysis. To validate these findings, future studies should consider employing membrane-potential-sensitive dyes like DiBAC₄(3) or high-resolution electron microscopy, which can offer direct visual confirmation of structural damage (Wang, Bai, et al., 2023).

### 4.3 DNA interaction and inhibition

Beyond inducing oxidative stress and compromising membrane integrity, certain phenolic compounds exert direct genotoxic effects by interacting with bacterial DNA—either through physical binding to nucleic acids or disruption of transcriptional machinery. These interactions may hinder DNA replication and restrict enzyme accessibility, thereby contributing to sustained antibacterial activity that extends beyond immediate cellular damage (Peng et al., 2025).

**Fig. 5:**
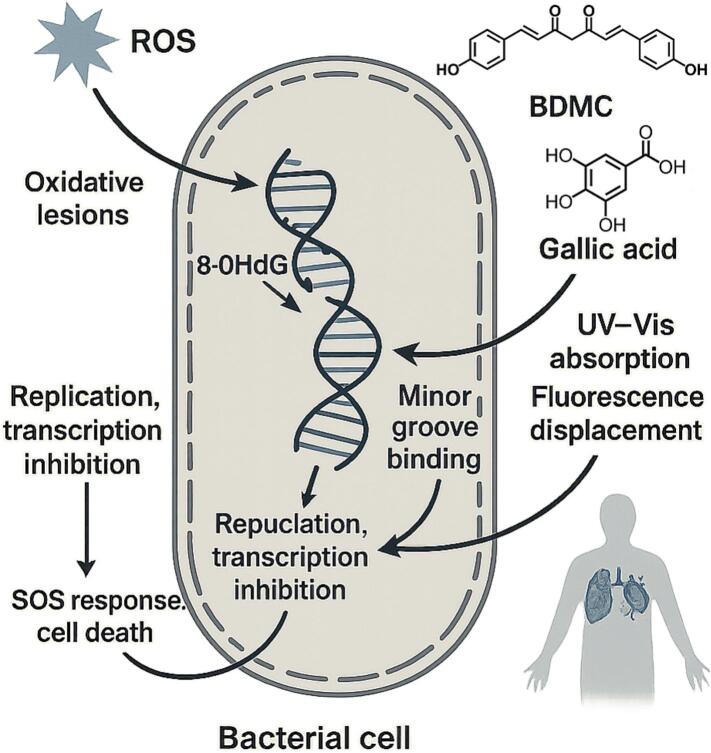
Mechanistic representation of natural phenolic compounds interfering with bacterial DNA. BDMC and gallic acid (structures, upper right) penetrate the cell and (i) bind within the DNA minor groove, verified by UV–vis absorption shifts and fluorescence displacement assays, and (ii) promote reactive‑oxygen-species (ROS) formation, generating oxidative lesions such as 8-OHdG. Structural distortion and oxidative damage inhibit replication and transcription, trigger an SOS stress-response cascade, and culminate in cell death.

Natural phenolics such as BDMC and gallic acid have been shown to interfere with bacterial DNA via two complementary mechanisms: oxidative lesions induced by ROS and direct binding to the DNA helix. As illustrated in Fig. 5, ROS generated upon BDMC treatment may cause base modifications such as 8-hydroxy-2′-deoxyguanosine (8-OHdG), while BDMC molecules can insert into the DNA minor groove, ultimately impairing replication and transcription.

**Fig. 6:**
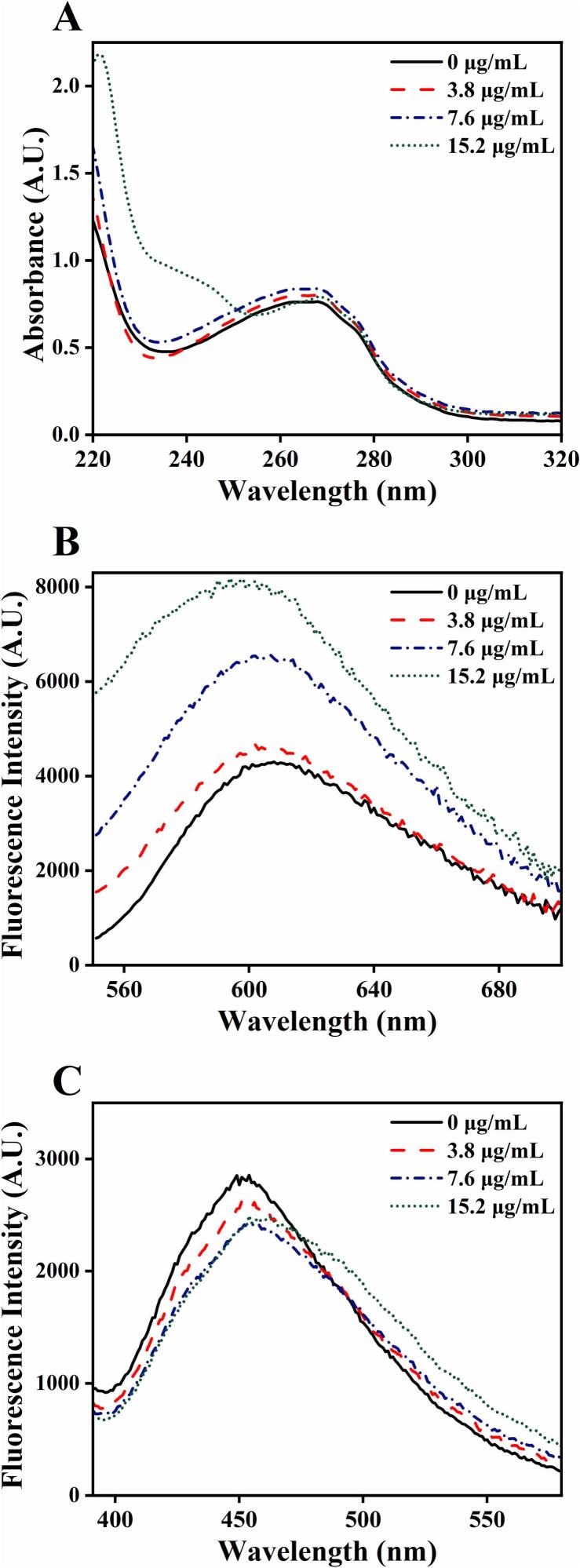
Spectroscopic assessment of BDMC binding to *Escherichia coli* genomic DNA. (A) UV–Vis spectra showing a dose-dependent hyperchromic effect at ≈260 nm with no detectable red shift in λ_max_, consistent with groove binding rather than classical intercalation. (B) Enhancement of ethidium bromide (EB)–DNA fluorescence in the presence of BDMC, plausibly due to helix loosening that facilitates intercalation. (C) Dose-dependent quenching of DAPI (4′,6-diamidino-2-phenylindole)–DNA fluorescence, indicating minor-groove competition and/or BDMC-induced structural perturbation. Values are mean ± SD (*n* = 3). Representative laboratory data (raw data available upon request). *EB, ethidium bromide; DAPI, 4′,6-diamidino-2-phenylindole; a.u., arbitrary units.* (For interpretation of the references to colour in this figure legend, the reader is referred to the web version of this article.)

DNA interaction. Fig. 6 shows spectroscopic evidence consistent with a DNA-targeting mechanism, with raw data available upon request. UV–visible absorption spectra revealed a concentration-dependent hyperchromic effect at ≈260 nm after BDMC addition, without a red shift in λ_max, supporting groove binding rather than intercalation (Fig. 6A). Fluorescence intercalator-displacement assays further showed that BDMC enhanced EB–DNA fluorescence (Fig. 6B) yet quenched DAPI–DNA fluorescence in a dose-dependent manner (Fig. 6C), indicating minor-groove competition and/or structural perturbation of genomic DNA. Findings are in line with published observations (Ghosh et al., 2018).

Together, these findings suggest that BDMC binds to bacterial DNA via a non-intercalative, groove-binding mode while simultaneously modulating helical conformation—as summarised in Fig. 6. This dual-mode behavior is consistent with reports on other polyphenolic antimicrobials that associate with DNA and interfere with transcriptional processes through structural alteration (Bag et al., 2025).

Nonetheless, limitations must be acknowledged. In vitro fluorescence-based assays lack chromatin complexity and may be influenced by probe-specific artefacts. Future studies incorporating circular dichroism, surface plasmon resonance, or isothermal titration calorimetry are warranted for validation (Mahendrawada et al., 2025).

In summary, BDMC exhibits concentration-dependent DNA-binding properties that complement its ability to generate ROS and disrupt membranes. This multifaceted mechanism underpins its broad-spectrum antibacterial efficacy.

## 5 Factors affecting efficacy in food systems

While natural phenolic compounds demonstrate promising antibacterial activities under laboratory conditions, their efficacy in real food matrices is frequently modulated by a range of extrinsic and intrinsic factors. Understanding these influences is essential for translating mechanistic insights into practical applications in food preservation (Chen et al., 2024).

### 5.1 Representative food models and effective concentrations

Natural phenolics are now being tested under process-relevant conditions in a range of foods (Table 2). Their efficacy, however, is matrix-dependent, being shaped by pH, fat level, water activity and resident microbiota, all of which modulate phenolic solubility, diffusion and target accessibility.

**Table 2:** Application of representative natural phenolic compounds in real food systems: effective concentrations, target pathogens, and antibacterial outcomes.

Food Model	Phenolic Compound	Dose	Target Organism(s)	Log Reduction	Storage Conditions	Ref
Pork patties	Thymol	250 μg/g	*Listeria monocytogenes*	∼2.5 log (7 d)	4 °C	Hashemi et al. (2023)
Apple juice	Curcumin + UV-A (aPDT)	1–10 mg/L	*E. coli* O157:H7	5 log (< 15 min)	Room temperature	Baskaran et al. (2010); Falcao de Oliveira et al. (2018)
Yogurt	Rosemary essential oil	0.5–0.7 % v/w	Total viable count, yeasts, molds	Delayed spoilage; yeast growth inhibited up to 12 d	4 °C	Ali et al. (2021); Kamel et al. (2022)
Shrimp	Green tea extract (rich in EGCG)	–	*Vibrio parahaemolyticus*	Significant inhibition (log value not specified)	–	Coelho et al. (2021)

In fresh pork patties, thymol incorporated at 250 μg/g reduced *Listeria monocytogenes* by ≈2.5 log after seven days of chilled storage (4 °C), without compromising colour or flavour attributes (Hashemi et al., 2023). Photodynamically activated curcumin offers an alternative for clear beverages: aPDT treatment of apple juice with 1–10 mg/L curcumin plus UVA light achieved a 5 log reduction of *E. coli* O157:H7 within 15 min at ambient temperature (Baskaran et al., 2010; Falcao de Oliveira et al., 2018).

Dairy matrices present an additional challenge because fat and casein can bind phenolics. Even so, 0.5–0.7 % (*v*/*w*) rosemary essential oil delayed yeast and mould spoilage in stirred yogurt for up to 12 days at 4 °C, while sustaining starter-culture viability—an effect attributed to the oil's phenolic diterpenes acting at the cream–serum interface (Ali et al., 2021; Kamel et al., 2022).

Seafood applications remain less explored. A green-tea extract rich in EGCG applied to ice-stored shrimp curtailed the growth of *Vibrio parahaemolyticus*; although the original study did not report exact log reductions, plate counts were consistently lower than untreated controls throughout chilled storage (Coelho et al., 2021). The result highlights the promise of catechin-based interventions in high-water, high-protein systems where many essential oils fail because of rapid volatilisation.

Despite these successes, phenolic activity in foods is typically lower than in broth: protein binding, lipid partitioning and buffering effects can mask up to 90 % of the in vitro potency. Current formulation strategies therefore focus on nano- or micro-encapsulation, mild heat or light activation, and synergistic blends (e.g., phenolics with low-level organic acids) to preserve antimicrobial performance while meeting sensory expectations. Continued work on matrix-specific delivery will be critical to translate the multitarget mechanisms discussed earlier into robust, industry-ready solutions.

### 5.2 Food matrix complexity

The heterogeneous composition of food systems—containing proteins, lipids, carbohydrates, and water—can significantly alter the bioactivity of phenolic compounds. Proteins and polysaccharides may bind phenolics via hydrogen bonding or hydrophobic interactions, thereby reducing the free, active fraction available to target bacterial cells (Günal-Köroğlu et al., 2023). Lipid-rich environments may also sequester hydrophobic phenolics like BDMC, limiting their diffusion and bioavailability.

### 5.3 Environmental parameters (pH, temperature, and water activity)

pH can affect both the ionization state and stability of phenolic compounds. BDMC, for example, exhibits reduced solubility and increased aggregation in acidic environments, potentially compromising its cellular uptake and ROS-inducing ability (Wang, Cai, et al., 2024). Similarly, temperature can influence phenolic–target interactions, enzymatic degradation, and membrane fluidity in bacteria, thereby modulating antimicrobial efficacy.

Low water activity (aw) environments, common in dried or intermediate-moisture foods, may reduce the diffusion of phenolics and limit bacterial metabolism, thereby masking or diminishing the observable antibacterial effect (Zamuz et al., 2021). Therefore, antimicrobial performance must be evaluated under matrix-specific conditions, rather than extrapolated from in vitro data alone.

### 5.4 Interaction with food microbiota

In real food systems, phenolic compounds interact with both target pathogens and background microbiota. Sub-lethal exposure may induce stress responses or tolerance mechanisms in bacteria, including upregulation of efflux pumps or antioxidant defenses (Ahlawat et al., 2024). Furthermore, commensal microorganisms may metabolize phenolics, reducing their effective concentration or altering their chemical structure. These microbial transformations are seldom observed in simplified culture models but may critically affect antibacterial outcomes in practice (López de Felipe et al., 2022).

### 5.5 Delivery systems and formulation strategies

To overcome these limitations, various delivery platforms—such as nanoemulsions, biopolymer encapsulation, and active packaging films—have been proposed to enhance the stability, solubility, and targeted release of phenolic antimicrobials in food systems (Wang, Dekker, et al., 2024). These strategies may also protect phenolics from premature degradation, preserve their multitargeting properties, and enable controlled, surface-localized release in foods that enhances contact killing—via ROS-mediated damage and membrane disruption—upon microbial contact (Confessor et al., 2024; Xiao et al., 2023).

Taken together, the performance of multitarget phenolic antimicrobials such as BDMC is context-dependent and must be evaluated within the physicochemical and microbial complexity of food environments. Further studies are needed to correlate molecular-level mechanisms with antimicrobial outcomes under realistic processing and storage conditions.

## 6 Limitations and data transparency

To contextualize the evidence synthesized in this review, we first outline key limitations of the current literature (Section 6.1) and then detail data transparency and availability for the illustrative datasets shown in Fig. 5, Fig. 6 (Section 6.2).

### 6.1 Limitations of the review

The evidence base remains heterogeneous across pathogens, food matrices, and assay formats, limiting direct comparability of effect sizes. Many mechanistic reports are conducted in vitro and may not fully capture constraints imposed by real foods (pH, a_w, fat, proteins, and light exposure). Endpoints and probes for ROS, membrane damage (e.g., PMF depolarization, permeability), and DNA interaction are not yet standardized across laboratories, and publication bias toward positive findings cannot be excluded. Temporal sequencing and the relative contribution of each target in multitarget phenolics are still incompletely resolved under industrially relevant storage and illumination conditions. Finally, coverage is uneven across compound classes, with abundant data for a few representatives (e.g., EGCG, thymol/carvacrol) but limited evidence for others (e.g., BDMC and less-studied phenolics) in specific food systems.

### 6.2 Data transparency and availability

In addition to the published literature synthesized herein, Fig. 5, Fig. 6 include illustrative laboratory datasets generated in 2023–2024 under standardized and reproducible conditions. These datasets are provided solely as mechanistic exemplars and are interpreted alongside the published evidence cited in 3, 4, 5. Raw data (e.g., fluorescence traces for PI/DiSC3(5) and DCFH-DA, UV–Vis spectra, and viability readouts) are available from the corresponding author upon reasonable request and can be supplied to editors or reviewers for verification. Potential sources of measurement bias (dye artefacts, instrument drift, inner-filter effects) were mitigated through appropriate controls (vehicle, dye-only, cell-only), calibration, and biological triplicates (*n* ≥ 3). These transparency measures are intended to complement the literature and to illustrate specific multitarget mechanisms in a food-relevant context without overstating the generality of any single dataset.

## 7 Outlook

In light of the tripartite framework outlined in this review—namely, reactive oxygen species generation, membrane integrity disruption, and DNA interaction—natural phenolic compounds emerge as versatile antibacterial agents with considerable potential for food preservation. However, several scientific and practical challenges remain unresolved. These include the mechanistic hierarchy of target effects, the translational gap between in vitro and in situ efficacy, and the regulatory constraints governing food applications. Addressing these gaps requires an integrated research agenda encompassing mechanistic studies, formulation strategies, and compliance with safety and labeling regulations.

### 7.1 Mechanistic elucidation and target prioritisation

Although multitarget phenolic antimicrobials have shown promising in vitro performance, the precise temporal sequence and relative contribution of each antibacterial mechanism remain unclear. Advanced techniques such as transcriptomics, live-cell imaging, and redox proteomics could help disentangle the causal pathways linking ROS generation, membrane disruption, and DNA interference. In particular, it remains to be determined whether certain targets dominate depending on bacterial species, growth phase, or food matrix conditions (Hu et al., 2024). Unraveling these context-dependent hierarchies will be essential for tailoring phenolic combinations with maximal synergistic potential.

### 7.2 Delivery strategies and food system integration

The efficacy of phenolic antimicrobials is frequently reduced in real food matrices due to limited solubility, volatility, and interactions with proteins or lipids. Novel delivery technologies—such as nanoemulsions, biopolymer encapsulation, and active coatings—offer promising avenues to preserve antimicrobial activity while minimizing sensory alterations (Wang, Dekker, et al., 2024). However, successful integration into food systems requires a balance between efficacy, stability, cost, and consumer acceptance. Studies focusing on real-time performance under industrial storage and distribution conditions remain scarce and should be prioritized.

### 7.3 Regulatory landscape and safety considerations

The commercial use of natural phenolics in food preservation is subject to region-specific regulatory frameworks concerning safety, dosage, and labeling. The regulatory frameworks across major jurisdictions are summarised in Table 3. In the EU, the European Food Safety Authority (EFSA) includes rosemary extract (E 392) among approved additives with specific concentration limits, such as ≤400 mg/kg in fats and oils (Additives et al., 2018). In the U.S., the Food and Drug Administration (FDA) classifies several phenolic compounds—such as eugenol and thymol—as Generally Recognized as Safe (GRAS) when used under defined technical conditions (Saini et al., 2025). The regulatory frameworks across major jurisdictions are summarised in Table 3.

**Table 3:** Regulatory status of selected natural phenolic antimicrobials across major food markets.

Region	Regulatory Body	Approved Phenolic Ingredients	Approval Route	Example Limit
EU	EFSA	Rosemary extract (E 392)	Food additive	400 mg/kg in fats and oils
US	FDA	Eugenol, Thymol, Gallic acid	GRAS	Case-specific (e.g., Eugenol ≤ 0.02 % in chewing gum; Gallic acid ≤ 0.1 % in beverages)
China	NHC	Tea polyphenols, GSP	Food additive	0.02–0.1 % (by weight, depending on food category); tea polyphenol ester max. 0.06 % in fats & oils

Note: Maximum permitted levels (MPLs) for rosemary extract (E392) are taken from EFSA (2018) and Commission Regulation (EU) No 1333/2008; 400 mg/kg applies to fats, oil emulsions, and savory fat spreads. GRAS usage levels are application-specific. For example, eugenol is permitted up to 0.02 % in chewing gum (FEMA 2467), and gallic acid up to 0.1 % in beverages (FDA GRN 000686). Tea polyphenols and grape seed proanthocyanidins (GSP) are permitted as food additives in China under GB 2760 standards, with maximum levels ranging from 0.02 to 0.1 % depending on food category. For instance, tea polyphenol palmitate is allowed at up to 600 mg/kg in oils and fats.

In China, the regulatory framework for food additives—particularly those derived from natural phenolic compounds—is governed by the National Health Commission (NHC) through GB 2760-2014 and its subsequent revisions. According to this standard, tea polyphenols (INS No. 343ii) are approved as antioxidant agents for use in various food categories, with maximum allowable concentrations ranging from 0.01 to 0.1 g/kg, depending on the food matrix, such as beverages or instant noodle seasoning packets. Notably, tea polyphenol palmitate is permitted at levels up to 600 mg/kg in oils and fats, reflecting its stability and lipid-solubility advantages [GB 2760-2014/2024].

Similarly, eugenol (CAS No. 97-53-0) is listed as a natural flavoring substance, regulated under the general use principle “QS” in GB 2760, meaning its dosage must follow the principle of “quantum satis” and good manufacturing practice rather than a fixed numerical limit. This flexible framework underscores the accepted use of naturally occurring phenolics in flavoring applications, provided safety and sensory thresholds are respected [GB 2760-2014/2024].

In contrast, complex botanical extracts—such as those derived from rosemary, cloves, or grape seeds—require standardization for active compound content. If not previously cataloged, they may be subject to toxicological assessment and pre-market approval under the “Measures for the Administration of New Food Additives” (2021 revision), 2021, particularly when proposed for use as preservatives or functional ingredients [NHC, 2021].

It is also important to recognize that labeling obligations differ based on application route. Phenolic antimicrobials incorporated into edible coatings, active packaging, or intelligent labeling systems may fall under separate functional classifications (e.g., food contact substances or food-related products), each with specific compliance requirements. In certain cases, allergen declarations may be mandatory if the phenolic source plant is known to trigger hypersensitivity reactions (Orhotohwo et al., 2024).

As regulatory priorities continue to shift toward clean-label formulations and minimally processed foods, there is an urgent need for harmonized toxicological standards and cross-border regulatory convergence (FAO/WHO, 2023). Establishing standardized dossiers for natural phenolic antimicrobials—covering their composition, safety, function, and application scope—will be essential for facilitating international market entry and ensuring consumer protection.

## 8 Conclusion

Natural phenolic compounds represent a multifunctional class of antimicrobial agents with growing relevance to food safety and preservation. This review proposes an integrated cascade model that links ROS generation, membrane disruption, and DNA interaction into a unified antibacterial mechanism. By consolidating evidence from 158 studies and supporting key mechanistic nodes with representative experimental data, the review provides a structured framework for understanding how multitarget actions reinforce each other to achieve enhanced efficacy.

Phenolics such as BDMC, thymol, and EGCG exemplify this multitarget behavior, offering broad-spectrum activity and reduced risk of resistance. However, their effectiveness in real food systems remains limited by bioavailability, stability, and matrix interference. To overcome these barriers, advances in delivery technologies—including nanoencapsulation, multilayer emulsions, and intelligent packaging—must be aligned with regulatory harmonization and safety validation.

Moving forward, greater emphasis should be placed on validating these mechanisms under realistic processing conditions and integrating phenolic antimicrobials into clean-label food preservation strategies. As outlined in Table 3, region-specific regulatory approvals already exist for selected phenolic antimicrobials, supporting near-term translation, whereas others will require additional safety packages and exposure assessments. The cascade model outlined in this review may also serve as a conceptual basis for designing synergistic formulations and guiding structure–function studies of next-generation natural preservatives.

## CRediT authorship contribution statement

**Lei Zhao:** Writing – original draft, Investigation. **Ya Zhou:** Methodology, Data curation. **Weiguo Yue:** Writing – original draft. **Qingshan Shen:** Writing – review & editing, Visualization. **Jingxuan Ke:** Methodology. **Yanli Ma:** Methodology. **Lifang Zhang:** Validation. **Hua Bian:** Methodology, Data curation.

## Declaration of competing interest

The authors declare that they have no known competing financial interests or personal relationships that could have appeared to influence the work reported in this paper.

## Data Availability

The datasets generated during the current study are available from the corresponding author on reasonable request.

## Appendix A Supplementary data

Supplementary material 1 PRISMA flow diagram of study identification, screening, and inclusion for antibacterial mechanisms of natural phenolic compounds (January 2013 – March 2025). Supplementary material 1

Supplementary material 2 Representative MIC/MBC values of phenolic compounds, methods checklist for key mechanistic assays, and data availability statement. Supplementary material 2
