# Supplementary material for: Natural phenolics as multitarget antimicrobials for food preservation: mechanisms of action
Source: Food Chem X. 2025 Sep 20;31:103056. doi: 10.1016/j.fochx.2025.103056 (PMC12495347; doi:10.1016/j.fochx.2025.103056)
Supplement: Supplementary material 1 — PRISMA flow diagram of study identification, screening, and inclusion for antibacterial mechanisms of natural phenolic compounds (January 2013 – March 2025). [file mmc1.docx]

**Figure_S1_PRISMA_FlowChart.**


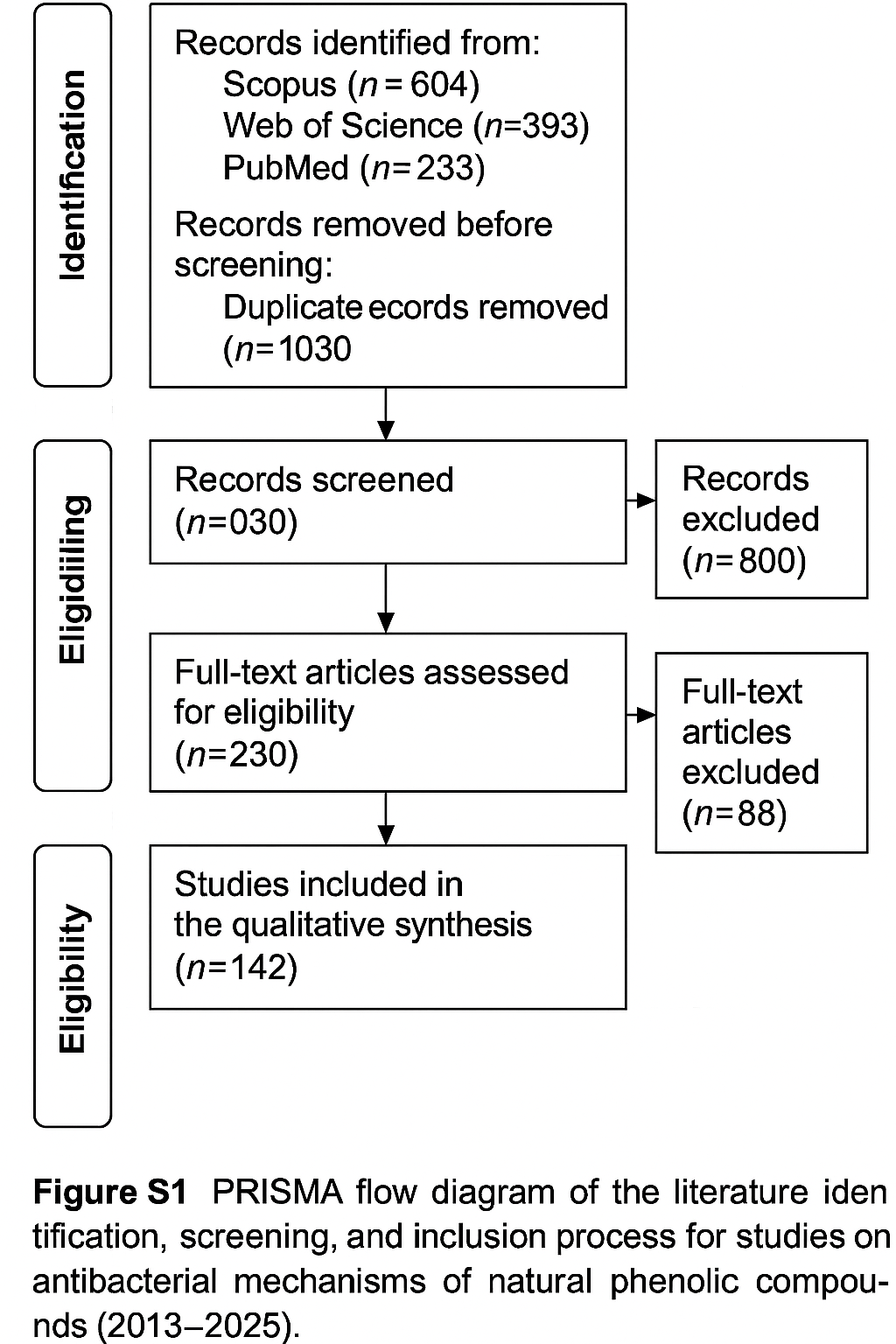


**Figure S1.** PRISMA flow diagram summarising the identification, screening, and inclusion of studies on antibacterial mechanisms of natural phenolic compounds (January 2013 – March 2025). A total of 1,230 records were retrieved (Scopus 604; Web of Science 393; PubMed 233). After removal of 200 duplicates, 1,030 unique records underwent title and abstract screening, during which 800 were excluded. Full-text assessment was performed for 230 articles; 88 were excluded owing to insufficient mechanistic data or non-foodborne strains. Consequently, 142 studies were included in the qualitative synthesis.
