# Supplementary material for: Natural phenolics as multitarget antimicrobials for food preservation: mechanisms of action
Source: Food Chem X. 2025 Sep 20;31:103056. doi: 10.1016/j.fochx.2025.103056 (PMC12495347; doi:10.1016/j.fochx.2025.103056)
Supplement: Supplementary material 2 — Representative MIC/MBC values of phenolic compounds, methods checklist for key mechanistic assays, and data availability statement. [file mmc2.docx]

**Supplementary Tables S2–S6**

Table S2. Search strategy and record counts (final update: 30 June 2025)

| **Database** | **Boolean search string (Title/Abstract/Keywords)** | **Date searched** | **Records retrieved** |
| --- | --- | --- | --- |
| Web of Science | TS = (“polyphenol*” OR “phenolic compound*”) AND TS = (antibacter* OR antimicrob*) AND TS = (“reactive oxygen species” OR ROS) AND TS = (membrane OR permeability) AND TS = (DNA OR genotoxic*) | 30 Jun 2025 | 393 |
| Scopus | TITLE-ABS-KEY (“polyphenol*” OR “phenolic compound*”) AND TITLE-ABS-KEY (antibacter* OR antimicrob*) AND TITLE-ABS-KEY (“reactive oxygen species” OR ROS) AND TITLE-ABS-KEY (membrane OR permeability) AND TITLE-ABS-KEY (DNA OR genotoxic*) | 30 Jun 2025 | 604 |
| PubMed | (“polyphenol”[TIAB] OR “phenolic compound”[TIAB]) AND (antibacterial OR antimicrobial) AND (“reactive oxygen species” OR ROS) AND (membrane OR permeability) AND (DNA OR genotoxicity) | 30 Jun 2025 | 233 |

Table S2. Database-specific search strings and document counts.

Table S3. Full-text articles excluded after eligibility assessment (n = 88)

| **No.** | **First author (year)** | **Abbreviated title** | **Reason for exclusion** |
| --- | --- | --- | --- |
| 1 | Zhang 2021 | Antioxidant activity of citrus peel flavonoids | No antibacterial endpoint |
| 2 | Kumar 2019 | Polyphenol nano-delivery in oncology | Non-foodborne application |
| 3 | Li 2020 | ROS modulation by quercetin derivatives | Mechanistic data absent |
| 4 | … | … | … |

*Table S3.* Summary of the 88 full-text articles excluded at the eligibility stage.

Table S4. Representative MIC/MBC values extracted from included studies†

| **Compound** | **Target strain** | **MIC (µg mL⁻¹)** | **MBC (µg mL⁻¹)** | **Assay medium** | **Ref. No.** |
| --- | --- | --- | --- | --- | --- |
| Bisdemethoxycurcumin | *E. coli* ATCC 25922 | 10 | 20 | Mueller–Hinton broth | 17 |
| EGCG | *E. col*i O157:H7 | 25 | 50 | LB broth | 23 |
| Thymol | *S. aureus* ATCC 6538 | 8 | 16 | TSB | 41 |
| Gallic acid | *L. monocytogenes* Scott A | 100 | 200 | BHI | 58 |
| … | … | … | … | … | … |

†The complete, unabridged dataset for all 142 studies is provided in *RawData_Fig5-6.xlsx* (Sheets “MIC-*Ecoli*” and “MIC-*Saureus*”).

*Table S4.* Minimum inhibitory (MIC) and bactericidal (MBC) concentrations of selected phenolic compounds.

Table S5. Methods checklist for key mechanistic assays

| **Assay** | **Key parameter** | **Setting / Instrument** |
| --- | --- | --- |
| DCFH-DA ROS assay | Probe concentration | 10 µM |
|  | λ<sub>ex</sub>/ λ<sub>em</sub> | 485 / 535 nm (SpectraMax iD5) |
| Propidium iodide permeability | Dye concentration | 10 µg mL⁻¹ |
|  | λ<sub>ex</sub>/ λ<sub>em</sub> | 530 / 620 nm (CLARIOstar Plus) |
| UV–Vis DNA binding | Scan range | 220–400 nm, 1 nm step (Cary 60) |
| Fluorescence displacement | EB concentration | 5 µM EB-DNA complex |
|  | Competitive ligand | 0–50 µM phenolic |

*Table S5.* Experimental settings for the principal mechanistic assays reported by the included studies*.*

Table S6. Data availability and risk disclosure statement

| **Item** | **Statement** |
| --- | --- |
| Unpublished data in main text | Figure 5 and Figure 6 include in-house experimental data generated in 2023–2024. |
| Raw data location | All primary fluorescence and permeability values are provided in RawData_Fig5-6.xlsx. |
| Access policy | The complete dataset will be supplied to editors and reviewers upon request and can be made publicly available after publication. |
| Conflict of interest | The authors declare no competing financial interests. |

*Table S6.* Transparency statement for unpublished data included in the review.
